# Isotopic and Confinement Effects on the Phase Behavior and Molecular Dynamics of Protonated and Deuterated Terphenyls in Electrospun Fibers

**DOI:** 10.1021/acs.jpcb.5c07839

**Published:** 2026-02-20

**Authors:** Anna Drzewicz, Szymon Folta, Olga Adamczyk, Marta Żak, Ewa Juszyńska-Gałązka

**Affiliations:** † Institute of Nuclear Physics Polish Academy of Sciences, PL-31342 Krakow, Poland; ‡ Faculty of Materials Science and Physics, Cracow University of Technology, PL-30084 Krakow, Poland; § Institute of Chemistry, Military University of Technology, PL-00908 Warszawa, Poland; ∥ Research Center for Thermal and Entropic Science, Graduate School of Science, Osaka University, 560-0043 Osaka, Japan; □ Institut für Angewandte Physik, Universität Tübingen, 72076 Tübingen, Germany

## Abstract

This work provides the first systematic analysis of isotopic effects on liquid crystal dynamics under two-dimensional polymer confinement. This study investigates the physicochemical properties and molecular dynamics of a partially fluorinated liquid-crystalline terphenyl and its deuterated analogue. Differential scanning calorimetry, polarized optical microscopy, and broadband dielectric spectroscopy are employed to characterize the mesomorphic behavior, thermal transitions, and relaxation dynamics of the compounds, both in bulk and confined within electrospun poly­(ε-caprolactone) fibers. While both isotopologues exhibit similar mesophases, the deuterated compound uniquely undergoes cold crystallization, highlighting the effect of deuteration on crystallization dynamics. When embedded in polymer fibers, two distinct glass transition temperatures reveal a heterogeneous, microphase-separated morphology. The incorporation of liquid crystals disrupts the crystalline structure of the polymer, increases the free volume, and reduces the activation energy of the β-relaxation process, especially for the deuterated variant. These effects point to the formation of dynamic microdomains with differentiated segmental and local mobility. The findings provide insight into how molecular confinement and isotopic substitution influence hybrid fiber system thermal and dielectric behavior, offering a design strategy for tunable materials in soft electronics and smart textiles.

## Graphical abstract

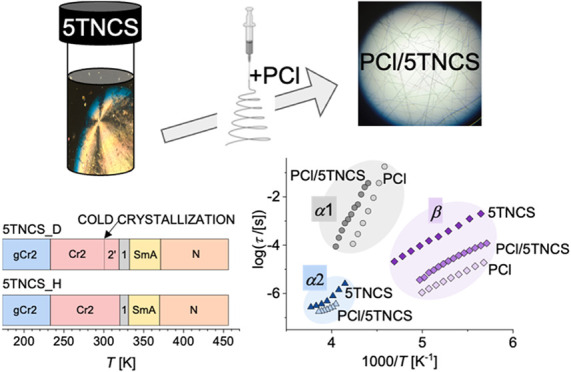

## 1 Introduction

Liquid crystals combine long-range order with fluidity due to their anisotropic molecular architecture. Typically, they consist of a rigid aromatic core and flexible terminal chains that control the mesophase stability. Liquid crystals display unique properties determined by their molecular anisotropy and internal structure. Numerous studies have shown that subtle structural modifications, such as fluorination, chain length variation, or linker substitution, can drastically alter mesomorphic behavior. Para-terphenyls remain a benchmark family for exploring the structure–property relationship in thermotropic systems. *Para*-terphenyls have attracted significant interest in structural research and conformational analysis owing to their conjugated π-electronic system, which arises from the delocalization of electrons within the terphenyl component.^1^ It has been demonstrated that the number and position of fluorine atoms in the molecular core significantly affect the tendency to crystallize or undergo a glass transition.^2^ Type of linking bridge affects the polymorphism of mesophases.^3^ Incorporating a −CH_2_O- linker into the molecule promotes antiferroelectric alignment of smectic layers,^4^ whereas a reduced number of −COO- linkers enhances chemical stability.^5^ Terphenyls with shorter alkyl chains (up to the pentyl group) are nematogens, while an elongation of the alkyl chain length results in the formation of smectic phases.^6^ Unbranched alkyl chains are the most typical terminal substituents. At the same time, the weakest bond in such structures is the C–C bond between Cα and Cβ (see Figure 1). The weakness of this bond arises from the stability of the product of its breakdown, which is the corresponding derivative of a benzyl radical, benzyl carbocation, or benzyl cation radical, all of which are strongly stabilized by resonance. It has been shown that replacing hydrogen atoms with deuterium atoms at the α and β positions increases the chemical stability of the entire molecule.^7^ Additionally, introducing deuterium at these positions is not expected to significantly affect the mesomorphic properties of liquid crystals.^8^ Instead, isotopic substitution primarily influences molecular dynamics by modifying the vibrational zero-point energies and local mobility. Consequently, deuteration provides a sensitive probe of kinetic and relaxation processes, including glass formation and crystallization, without significantly perturbing the thermodynamic phase behavior. The use of deuterated compounds as materials with an extended lifespan and improved performance parameters has already been successfully demonstrated for ligands,^9^ OLED diodes,^10^ polymers,^11^ photovoltaic applications,^12^ and various other systems.^13,14^

**Figure 1:**
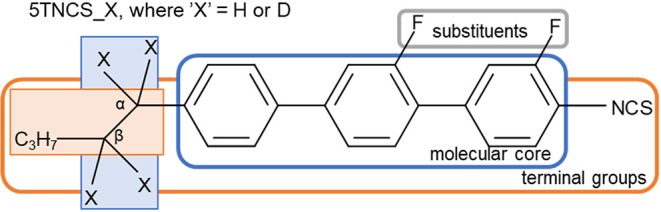
Formula of the investigated 5TNCS_X (where “X” = H or D) compounds with a typical mesogenic structure.

Despite decades of research on bulk terphenyls, the impact of spatial confinement on their molecular dynamics has not been systematically explored. The ongoing trend toward miniaturization has sustained significant interest in nanostructured systems. Accordingly, research into spatially confined environments is essential for developing materials with enhanced or novel properties. Numerous studies have explored the behavior of both organic and inorganic substances under conditions of nanoscale confinement.^15,16^ These investigations have consistently demonstrated that material properties at the nanoscale often diverge substantially from those observed in bulk systems. Nevertheless, despite considerable progress, a comprehensive understanding of how specific confinement types and degrees influence such systems’ physical and chemical behavior remains elusive. This challenge is especially pronounced for soft matter systems, including low- and high-molecular-weight glass-forming ionic liquids and liquid crystals.

Molecular confinement can occur in one, two, or three dimensions, across micro- or nanoscales, and under varying confinement rigidities (i.e., soft versus hard confinement).^17^ This leads to a wide range of architectures, such as thin films, nanoporous membranes, and fibers. Within such confined environments, molecules frequently exhibit physicochemical characteristics that markedly differ from those in their bulk counterparts, particularly under nanoconfinement where size-dependent phenomena dominate. As the system size diminishes, the surface-to-volume ratio increases, intensifying surface-related effects. Consequently, nanoscale materials often display unique properties that are absent in their macroscopic forms.

Despite growing interest in confinement phenomena, composite systems composed of electrospun fibers incorporating liquid crystals and polymers remain underexplored.^18^ Lagerwall et al.^19^ investigated electrospun fibers containing a nematic liquid crystal mixture, utilizing polarized Raman spectroscopy. Their findings revealed that such confinement can stabilize the nematic phase, analogous to the behavior observed in nanoporous systems. Electrospun fibers with embedded liquid crystals represent a promising class of functional materials, with potential applications in smart textiles^20^ and self-powered sensors.^21^

The first aim of this study is to examine the effect of deuteration on the physicochemical properties of partially fluorinated liquid-crystalline terphenyl and its deuterated isotopologue with deuterium atoms located at the α and β positions of the terminal alkyl chains. The compounds under study are 2′,3-difluoro-4-isothiocyanato-4″-pentyl-1,1′:4′,1″-terphenyl, abbreviated as 5TNCS_X (where “X” means H for protonated and D deuterated counterpart). The general chemical structure of the studied compounds is shown in Figure 1. In both compounds, the rigid core consists of a terphenyl group containing two fluorine atoms. One terminal chain is an isothiocyanate group (–NCS), while the compounds differ in the presence of hydrogen or deuterium atoms (“X” = H or D) at α and β positions of the terminal pentyl chains. For polymorphism studies, we used differential scanning calorimetry (DSC) and polarized optical microscopy (POM). DSC allows the description of thermodynamic parameters of phase transitions, and POM enables the characterization of mesophases based on the observed textures. For molecular dynamics studies, we used broadband dielectric spectroscopy (BDS), which provides information about the response of a dielectric material to an applied external electric field. Analyzing the physicochemical properties of difluorosubstituted terphenyl derivatives aligns with ongoing research into the dynamics of thermodynamic states in liquid crystals. The impact of terminal substituents, fluorination of alkyl chains, fluorination of the molecular core, the chiral terminal group, and the lateral halogens on liquid-crystalline properties for terphenyl-based molecules has been recently described. Systematic studies aimed at understanding how specific molecular structural components influence phase behavior (especially crystallization and/or glass transition) can contribute to the strategic design of liquid crystals with tailored properties for specific applications.

This study also focuses on the two-dimensional soft confinement of liquid crystal molecules within electrospun fibers. The fibers are fabricated from a biocompatible polymer, poly­(ε-caprolactone), PCL, and incorporate varying concentrations of the 5TNCS_X compound. To achieve a comprehensive understanding of the thermodynamic behavior and molecular-level dynamics, the fibers are systematically characterized using DSC for thermal analysis and BDS for dynamic studies. These techniques enabled the investigation of dielectric relaxation phenomena, molecular mobility, glass transition temperatures, and phase transitions within the liquid crystal-loaded fibers. As a result, valuable insight is gained into the molecular dynamics of 5TNCS_X under conditions of confinement within the fiber matrix.

Here, we investigate the influence of isotopic substitution (H/D) and soft confinement on the thermodynamic and dynamic behavior of a partially fluorinated terphenyl. Using differential scanning calorimetry, polarized optical microscopy, and broadband dielectric spectroscopy, we correlate phase transitions and relaxation dynamics with confinement and isotope effects. The results reveal distinct cold crystallization in the deuterated isotopologue and dual glass transitions in confined hybrid fibers, offering new insight into microphase-separated LC–polymer systems.

## 2 Experimental Section

### 2.1 Materials

The 5TNCS_X compounds were synthesized at the Institute of Chemistry, Military University of Technology, Warsaw, Poland (the general synthetic method for protonated 5TNCS_H is described in ref 22, and the synthetic method for deuterated 5TNCS_D is presented in ref 7). Poly­(ε-caprolactone) (PCL, average *M*_w_ = 80.000 g mol^–1^) was purchased from Sigma-Aldrich. Chloroform (≥99%) and methanol (≥99.8%) were obtained from POCH (Poland) and used as solvents in polymer solutions. All chemicals were analytical grade and used without further purification.

### 2.2 Electrospinning Technique

Composite fibers comprising PCL and varying concentrations of 5TNCS_X (0, 10, and 20 mg mL^–1^) were fabricated via the solution electrospinning method. The electrospinning precursor solution was prepared by dissolving 10 wt % PCL in a solvent system consisting of chloroform and methanol (75:25 v/v). The resulting fibers were collected on a square metallic collector plate. For electrospinning, an applied voltage of 8.5 kV was used for the pure PCL solution, while a reduced voltage of 7.0 kV was applied for the PCL–5TNCS_X mixtures. The distance between the 2 cm long hypodermic needle tip and the collector was maintained at 11 cm, with a constant flow rate of 1.5 mL h^–1^.

### 2.3 Experimental Methods

Differential scanning calorimetry (DSC) thermograms were registered by using a TA DSC 2500 calorimeter. The indium and sapphire standards were used for the calibration. The samples in the bulk form with the masses of 5.26 mg for 5TNCS_H and 4.76 mg for 5TNCS_D were cooled and heated with several rates (±2, ±5, ±10, ±15, ±20, ±25, and ±30 K min^–1^). The modulated-temperature (MT-DSC) regime measurements were performed at a heating rate of 3 K min^–1^ with a temperature modulation amplitude of 1 K and a modulation period of 60 s. MT-DSC provides deeper insights into the thermodynamic and kinetic aspects of phase transitions.^23^ The total heat flow, commonly measured in standard DSC, consists of two distinct components: the reversing heat flow, which reflects heat capacity, and the nonreversing heat flow, which captures the kinetic contribution. The samples in the fibers form were measured upon cooling and heating in the 173–345 K temperature range at rates of ±5, ±10, ±15, and ±20 K min^–1^. The data analysis was done in the TRIOS program. Phase transition temperatures, i.e., onset temperature (*T*_onset_), peak temperature (*T*_peak_), and glass transition temperature (*T*_g_), were estimated for each thermal anomaly. The *T*_g_ was determined as the midpoint of the heat capacity change using the half-extrapolated tangent method.

Polarizing optical microscopy (POM) textures were observed using a Leica DM2700P microscope with the crossed polarizers equipped with a Linkam LNP96-S heating/cooling stage and Linkam T96-S temperature controller. The samples in the bulk form were placed on covered glass plates without aligning layers in the isotropic liquid phase and were fast cooled and subsequently heated at a rate of 4 K min^–1^. The data analysis was done in the TOApy program.^24^

Broadband dielectric spectroscopy (BDS) measurements were performed using a high-resolution Novocontrol α Analyzer with the Novocontrol temperature controller, in the frequency range of 0.1 Hz–10 MHz. The samples in the bulk form were placed between two gold electrodes with an active area of 10 mm, without aligning layers, and with poly­(tetrafluoroethylene) spacers with a thickness of 75 μm. The samples in the fibers form were cut into squares and placed between two circular gold electrodes. Dielectric spectra were collected upon heating at a rate of 2 K min^–1^ after fast cooling. The data analysis was done using the WinFit program.

All of the above measurements were carried out under a nitrogen atmosphere in the temperature range from 173 K to the transition temperature to the isotropic liquid phase.

## 3 Results and Discussion

### 3.1 Phase Behavior of 5TNCS_X in the Bulk Form

Microscopic observations enable visualization of textures occurring in specific thermodynamic phases. They also facilitate the identification of individual phases by comparing the observed textures with catalogued reference patterns. Figure 2 shows textures recorded at different temperatures during heating of the 5TNCS_H sample at a rate of 4 K min^–1^. At 223 K, cracks characteristic of a glassy state are visible,^25^ specifically the glassy state of the crystalline phase (gCr2). The next image, taken at 273 K, corresponds to the crystalline phase Cr2, while at a higher temperature (323 K), the texture of the crystalline phase Cr1 is observed. At 353 K, a fan-shaped texture characteristic of the smectic A phase (SmA) is recorded. In the last image, recorded at 468 K, the phase transition from the isotropic liquid to the nematic phase (Iso-N) is observed.

**Figure 2:**
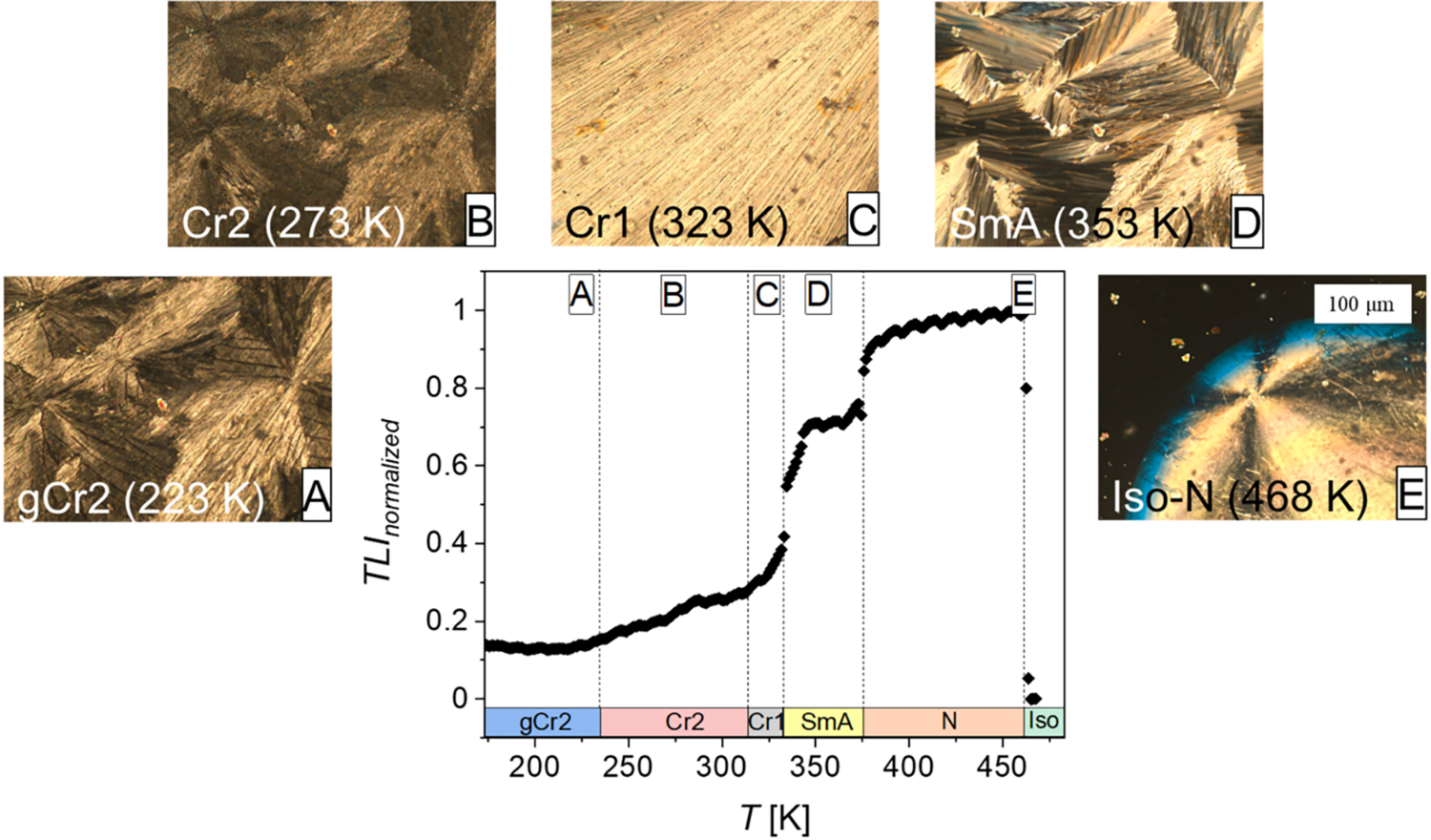
POM textures and TOA of the 5TNCS_H registered upon heating at a rate of 4 K min^–1^, with the marked thermodynamic phase range obtained from DSC.

The recorded textures are used to perform a thermo-optical analysis (TOA) using the TOApy software. The normalized TLI refers to the amount of light passing through the sample during heating or cooling. This value is normalized, meaning that the *Y*-axis was adjusted to range from 0 to 1. Jumps or inflection points visible on the TOA graph suggest the occurrence of phase transitions. An inflection point around 233 K indicates the gCr2-Cr2 transition. A flattening of the TLI curve near 308 K corresponds to the transition between the crystalline phases. A sharp increase in the TLI around 333 K is associated with the Cr1-SmA transition. Another sharp increase at approximately 373 K corresponds to the SmA-N transition. At around 463 K, the transition to the isotropic liquid phase (Iso) occurs, visible as a sudden decrease in TLI values.

To determine the precise phase transition temperatures and associated thermal effects, the results of the DSC analyses are evaluated. DSC thermograms recorded for chosen cooling and heating rates (±2 and ± 20 K min^–1^) of the 5TNCS_H and 5TNCS_D compounds are shown in Figure 3a,b. The determined phase transition temperatures (*T*_onset_ and *T*_peak_), as well as the corresponding changes in enthalpy (Δ*H*) and entropy (Δ*S*) for all cooling and heating rates (±2, ±5, ±10, ±15, ±20, ±25 and ±30 K min^–1^), are summarized in Supporting Information (Tables S1 and S2).

**Figure 3:**
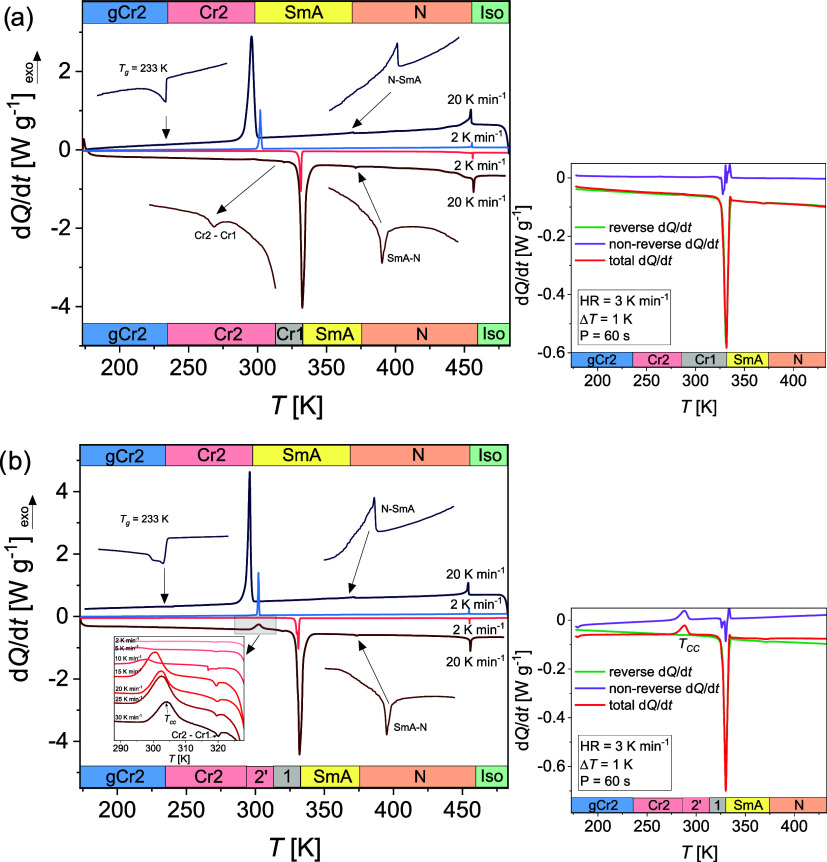
DSC thermograms of the 5TNCS_H (a) and 5TNCS_D (b) collected upon cooling (blue) and heating (red) rates (±2 and ± 20 K min^–1^). The insets show the glass transition temperature (*T*_g_), the N-SmA, Cr2–Cr1, and SmA-N transitions, and the cold crystallization temperature (*T*_cc_) for all heating rates. The right panel presents the MT-DSC plots performed at the heating rate (HR) of 3 K min^–1^ with the temperature modulation amplitude (Δ*T*) of 1 K and the modulation period (*P*) of 60 s.

The first anomaly, observed at approximately 456 K during the cooling of the sample, corresponds to the Iso-N transition, as evidenced by an entropy change below 3 J mol^–1^ K^–1^, consistent with values reported in ref 26. A small anomaly appears at approximately 369 K, associated with the N-SmA transition. This transition occurs between two liquid-crystalline mesophases, characterized by the lowest degree of molecular order among known mesophases, resulting in an entropy change of less than 0.5 J mol^–1^ K^–1^. The most pronounced anomaly is observed near 293 K and corresponds to the SmA-Cr2 transition. The entropy change below 60 J mol^–1^ K^–1^ indicates significant structural changes typical of molecular ordering. For kinetically controlled and nonequilibrium transitions, including SmA-Cr2 crystallization and cold crystallization, the entropy changes estimated from DSC measurements do not represent equilibrium state functions but serve as comparative measures of the extent of structural reorganization. The anomaly observed at approximately 233 K, probably associated with the glass transition (Cr2-gCr2), appears as a peak rather than a classical step-like change in the heat capacity typically associated with a glass transition. This behavior arises because only a subset of molecular degrees of freedom (orientational or conformational) undergo kinetic arrest, and the peak corresponds to enthalpy relaxation during cooling. Moreover, the presence of a glass-like transition suggests that the Cr2 phase cannot be regarded as a fully ordered crystal but rather corresponds to a dynamically disordered crystalline phase with residual molecular mobility. Upon heating, glass softening occurs at approximately 234 K. At higher heating temperatures, an exothermic anomaly is observed, only for 5TNCS_D, near 300 K (inset in Figure 3b), while all other anomalies during the heating cycle are endothermic. This anomaly is attributed to the process of cold crystallization, which occurs during heating from the glassy state, as opposed to melt crystallization, which takes place during cooling. For all heating temperatures below 323 K, an anomaly associated with the transition between the low-temperature crystalline phase (Cr2) and the high-temperature crystalline phase (Cr1) is observed. The entropy change for this transition is approximately 0.1 J mol^–1^ K^–1^, suggesting that the transition may primarily reflect lattice vibrational contributions. Below 333 K, an anomaly corresponding to the Cr1-SmA transition is observed, while the anomalies related to the SmA-N and N-Iso transitions occur at temperatures similar to those determined during the cooling cycle. The MT-DSC curves (insets in Figure 3a,b) show that the cold crystallization process is visible only on the irreversible heat flow curve for the 5TNCS_D. Additionally, the Cr1-SmA transition exhibits a complex behavior on this curve, indicating the thermodynamic irreversibility of these processes.

#### 3.1.1 Kinetics of Nonisothermal Cold Crystallization of 5TNCS_D

Typically, protonated compounds and their deuterated counterparts exhibit very similar mesomorphic behavior, including comparable phase sequences and transition temperatures. However, in the present case, cold crystallization is observed exclusively for the deuterated isotopologue 5TNCS_D, while the protonated analogue 5TNCS_H remains fully amorphous upon reheating. This behavior indicates that deuteration subtly modifies the free-energy landscape of the glassy state. The increased molecular mass and altered zero-point vibrational energies reduce the local molecular mobility, effectively stabilizing the amorphous state during cooling. Upon subsequent heating, this reduced mobility favors delayed but more efficient nucleation, leading to cold crystallization. In contrast, the higher molecular mobility of the protonated compound suppresses nucleation and prevents crystallization under identical thermal conditions.

To quantitatively describe this process, the kinetics of nonisothermal cold crystallization of 5TNCS_D were investigated using DSC measurements performed at different heating rates. The following analysis focuses on how molecular mobility and diffusion-controlled mechanisms govern crystallization upon heating from the glassy state.

The cold crystallization process, which involves crystallization upon heating from the glassy state, can occur according to classical thermodynamic predictions or via diffusion, which is linked to the mobility of the molecules. The occurrence of cold crystallization is influenced by the thermal history of the sample. To characterize this process under nonisothermal conditions, the sample is first cooled from the isotropic phase to the glassy state. Subsequently, the sample is heated, and DSC thermograms are recorded at various heating rates. The cooling rate prior to the heating cycle is consistent with the applied heating rate. For the 5TNCS_D compound, we observed the cold crystallization for heating rates between 5 K min^–1^ and 30 K min^–1^ (inset in Figure 3b).

The degree of crystallization (*D*) for the nonisothermal process can be determined for each heating rate (d*H*/d*T*) using the following formula^27^

1 $$D\left(T\right)=\frac{\int_{T_{b}}^{T}\left(\frac{dH}{dT}\right)dT}{\int_{T_{b}}^{T_{e}}\left(\frac{dH}{dT}\right)dT}$$

where *T*_b_ and *T*_e_ are the beginning and ending temperatures of crystallization. For 3D, the degree of crystallinity vs temperature curves are shifted toward higher temperatures with increasing heating rates (Figure 4a). To describe the kinetics of the nonisothermal cold crystallization, the Ozawa model is used, here in its logarithmic formula^28^

2 $$\log\left(-\ln\left(1-D\right)\right)=\log\left(Z\right)-n_{O}\log\left(dT/dt\right)$$

where *Z* is the Ozawa crystallization rate and *n*_O_ is the Ozawa exponent depending on the crystal size. In the linear Ozawa plot, the slope is related to *n*_O_, while the point of intersection with the OY axis indicates the value of the log­(*Z*), Figure 4b. The *n*_O_ parameter changes between 2.61 and 4.24, so the dimensions of the resulting crystals vary based on the heating rate applied to the sample, Figure 4c. The log­(*Z*) increases with increasing temperature, so the diffusion mechanism has an impact on the nonisothermal cold crystallization. The variations of the Ozawa parameters reveal distinct temperature-dependent transitions during the cold crystallization process. A noticeable increase in log­(*Z*) is observed at approximately 295 K, which coincides with the onset temperature (*T*_onset_) from the DSC, indicating the activation of molecular mobility and the initiation of nucleation. In contrast, a sudden change in *n*_O_ occurs at around 300 K, corresponding to the DSC peak temperature (*T*_peak_), suggesting a transition in the dominant crystallization mechanism from nucleation-controlled to growth-controlled. To determine the activation energy (*E*_A_) of this process, the Kissinger^29^ (eq 3) or Augis–Bennett^30^ (eq 4) may be used

3 $$\ln\left(\frac{dT/dt}{{T_{m}}^{2}}\right)=-\frac{E_{A}}{RT_{\max}}+\mathrm{const}$$

4 $$\ln\left(\frac{dT/dt}{T_{m}-T_{i}}\right)=-\frac{E_{A}}{RT_{\max}}+\mathrm{const}$$

where d*T*/d*t* is the heating rate, *T*_m_ is the maximum temperature of the anomaly, and *T*_*i*_ is the initial temperature. According to the linear dependences (Figure 4d), the activation energy of the nonisothermal cold crystallization is between 140 and 149 kJ mol^–1^.

**Figure 4:**
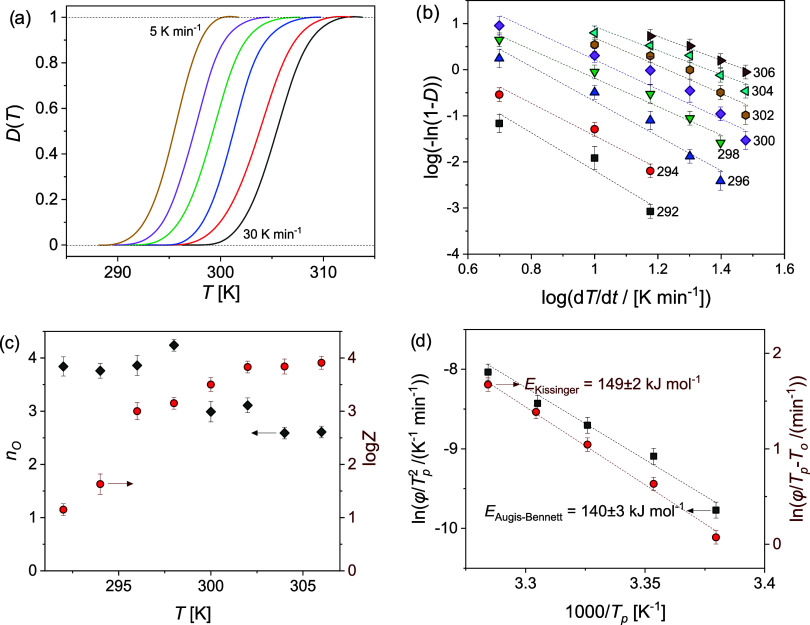
Temperature dependence of the crystallization degree (a), Ozawa plot (b), temperature dependence of Ozawa parameters (c), and Kissinger and Augis-Bennett plot (d) for the cold crystallization process of the 5TNCS_D.

The data are also analyzed using the isoconversional method, which enables the determination of the effective activation energy (*E*_eff_), as a function of *D* and *T* ^31^

5 $$\frac{dD\left(t\right)}{dt}=f\left(D\right)A\exp\left(-\frac{E_{\mathrm{eff}}}{\mathrm{RT}}\right)$$

where *f*(*D*) is the transition model and *A* is a preexponential factor. The logarithm of the crystallization rate ln (d*D*(*t*)/d*t*) is plotted against inverted temperature for selected crystallization degree 1000/*T*_D_ (Figure 5a) and *E*_eff_ is obtained from the slope of these activation plots. The values of *E*_eff_ are positive, indicating that the kinetics of the nonisothermal cold crystallizations is mainly controlled by the diffusion rate (Figure 5b).

**Figure 5:**
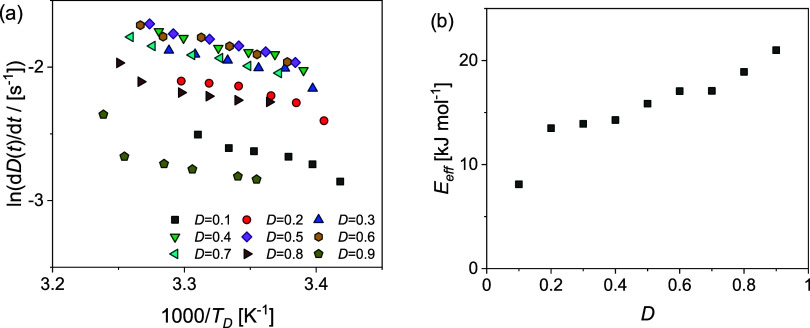
Activation plot based on isoconversional method for different *D* (a) and the effective activation energy vs crystallization degree (b) for the cold crystallization process of the 5TNCS_D.

These results demonstrate that deuteration alters the molecular mobility and the free-energy landscape in such a way that cold crystallization becomes energetically and kinetically accessible only in the deuterated compound.

### 3.2 Phase Behavior of 5TNCS_X Embedded within Electrospun Fibers

Poly­(ε-caprolactone), PCL, in its bulk form, exhibits a glass transition temperature of approximately 211 K and a relatively low melting point in the range of 328–333 K.^32^ The electrospinning process is known to reduce the degree of crystallinity, which suggests that the resulting electrospun fibers likely comprise both crystalline and amorphous regions of PCL and the liquid crystal. Consequently, the resulting material represents a complex hybrid system expected to display a combination of amorphous and crystalline characteristics, as evidenced in both DSC and BDS analyses. Additionally, the presence of distinct polymer and liquid crystal domains implies the potential observation of two separate glass transition events, provided that they fall within the detection limits of the applied techniques. The composite is most likely phase-separated and only partially miscible, indicating that the liquid crystal and polymer components do not form a uniform blend. Instead, the liquid crystal molecules appear to be physically entrapped within the fiber structure.

The phase behavior of the 5TNCS_X compounds in their bulk form is already well established. In the next stage, we investigated the phase situation of these compounds when embedded within electrospun fibers. The DSC thermograms of PCL fibers without liquid crystals (as a reference) and with 10 and 20 mg mL^–1^ 5TNCS_X content are presented for a cooling rate of 20 K min^–1^ (Figure 6) and the same heating rate (Figure 7), and the phase transition temperatures are shown in Table 1 (for cooling) and Table 2 (for heating).

**Figure 6:**
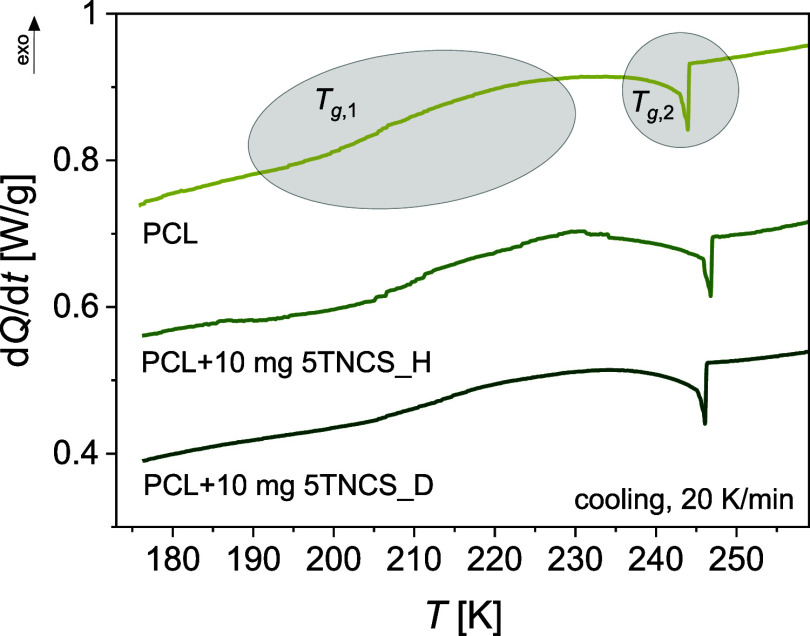
Comparison of DSC thermograms of fibers for cooling with a rate of 20 K min^–1^. The DSC thermograms are vertically shifted to improve clarity.

**Figure 7:**
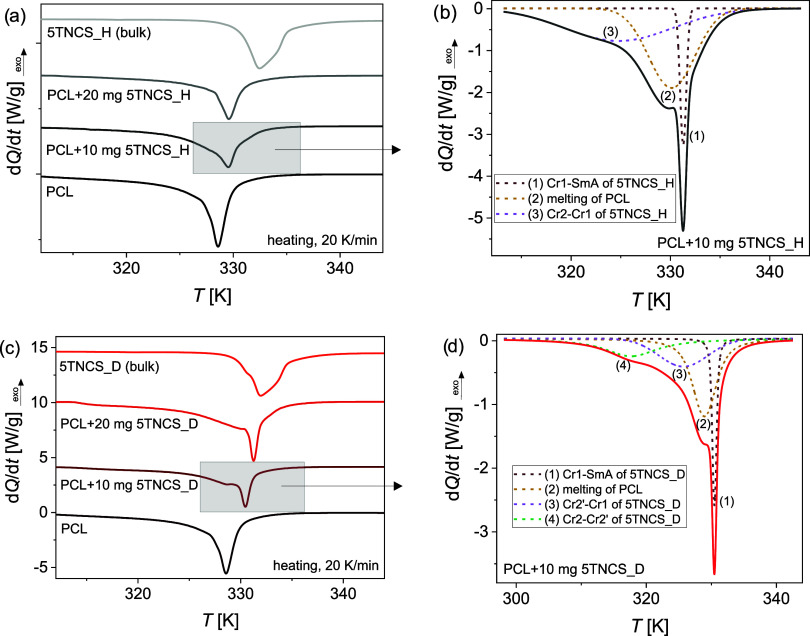
Comparison of DSC thermograms of samples in the bulk form and as fibers for 5TNCS_H (a) and 5TNCS_D (c) for heating with a rate of 20 K min^–1^. The enlarged area of crystallization and melting processes for 5TNCS_H (b) and 5TNCS_D (d). The DSC thermograms are vertically shifted to improve clarity.

**Table 1:** Glass Transition Temperatures (*T*_g,1_ and *T*_g,2_) Obtained for 20 K min^–1^ Cooling Rate

sample	*T*_g,1_ [K]	*T*_g,2_ [K]
PCL	208.8	245.1
PCL + 10 mg 5TNCS_H	216.1	247.0
PCL + 20 mg 5TNCS_H	220.9	246.3
5TNCS_H (bulk)		240.7
PCL + 10 mg 5TNCS_D	214.7	241.9
PCL + 20 mg 5TNCS_D	218.8	240.8
5TNCS_D (bulk)		233.3

**Table 2:** Phase Transitions Temperatures (*T*_p.t._) and Thermodynamic Functions (Enthalpy Change Δ*H* and Entropy Change Δ*S*) Obtained for 20 K min^–1^ Heating Rate^a^

sample	*T*_Cr2–Cr2′_ [K] / Δ*H* [kJ mol^–1^] / Δ*S* [J mol^–1^ K^–1^]	*T*_Cr2/Cr2′–Cr1_ [K] / Δ*H* [kJ mol^–1^] / Δ*S*[J mol^–1^ K^–1^]	*T*_PCLmelting_ [K] / Δ*H* [kJ mol^–1^] / Δ*S* [J mol^–1^ K^–1^]	*T*_Cr1–SmA_ [K] / Δ*H* [kJ mol^–1^] / Δ*S* [J mol^–1^ K^–1^]
PCL			328.7 / 7.7 / 23.4	
PCL + 10 mg 5TNCS_H		310.0 / 6.2 / 20.1	328.3 / 6.5 / 19.8	332.2 / 1.5 / 4.5
PCL + 20 mg 5TNCS_H		313.6 / 6.2 / 19.8	328.1 / 6.5 / 19.7	332.3 / 1.5 / 4.5
5TNCS_H (bulk)		319.9 / 0.04 / 0.14		332.1 / 20.4 / 61.4
PCL + 10 mg 5TNCS_D	306.9 / 3.8 / 12.3	321.0 / 3.6 / 11.3	325.3 / 6.4 / 19.7	332.4 / 2.4 / 7.3
PCL + 20 mg 5TNCS_D	307.0 / 3.8 / 12.3	322.3 / 3.6 / 11.2	324.7 / 6.4 / 19.6	332.0 / 2.4 / 7.3
5TNCS_D (bulk)	300.7 / 1.1 / 3.6	319.0 / 0.03 / 0.09		332.5 / 18.7 / 56.2

a Δ*H* and Δ*S* are expressed per mole of the liquid crystal.

Two distinct glass transitions are identified in the case of pure PCL fibers (Figure 6). The first, significantly broadened glass transition (*T*_g,1_), is observed at approximately 208.8 K, which is slightly lower than the reported value for bulk PCL (211 K).^33^ An additional, sharper glass transition (*T*_g,2_) appears at 245.1 K. Both the broadening of *T*_g,1_ and the emergence of a secondary *T*_g,2_ have been previously reported in the literature for PCL^34^ and other polymers subjected to spatial confinement, such as in thin films.^35^ For PCL + 5TNCS_X composite fibers, both *T*_g,1_ and *T*_g,2_ are shifted: *T*_g,1_ is observed in the range of 216.1–220.9 K for 5TNCS_H and 214.7–218.8 K for 5TNCS_D, while *T*_g,2_ appears at 247.0–246.3 K for 5TNCS_H and 241.9–240.8 K for 5TNCS_D (Table 1). Notably, an increase in the 5TNCS_X content correlates with an increase in *T*_g,1_. Given that no glass transition is observed for pure 5TNCS_X within this temperature range, *T*_g,1_ is attributed to the glass transition of the PCL phase. Conversely, *T*_g,2_ is present in both pure PCL fibers and pure 5TNCS_X, suggesting contributions from both components. The observed decrease in *T*_g,2_ with increasing 5TNCS_X content is likely due to the intrinsically lower *T*_g,2_ of the pure 5TNCS_X compounds compared with that of pure PCL.

The presence of two distinct glass transition temperatures (*T*_g,1_ and *T*_g,2_) in the DSC thermograms provides clear evidence of a microphase-separated and structurally heterogeneous system. *T*_g,1_ is associated with the segmental mobility of the PCL chains, whereas *T*_g,2_ reflects the combined thermal response of both PCL and the incorporated liquid crystal. This observation indicates partial miscibility between the polymer and the liquid crystal, leading to LC-rich and polymer-rich domains. Such microphase separation is characteristic of confined hybrid systems and has been reported for other LC–polymer composites under nanoscale confinement. The observed variations in glass transition behavior in the hybrid fiber systems are indicative of alterations in molecular segment dynamics. Specifically, the increase in *T*_g,1_ upon incorporation of 5TNCS_X suggests that the liquid crystal component may facilitate intermolecular interactions that restrict chain mobility. This effect is stronger for 5TNCS_H than that of its deuterated 5TNCS_D counterpart. Although *T*_g,1_ increases with higher concentrations of 5TNCS_X, *T*_g,2_ exhibits only a slight elevation upon liquid crystal incorporation and remains essentially invariant with further increases in its content.

Upon heating of the pure PCL fiber, its melting occurs at 328.7 K (Figure 7a). In the case of the composite fibers, a complex thermal anomaly is observed, comprising the Cr2–Cr1–SmA phase transitions of the 5TNCS_H compound and the melting of PCL (Figure 7b), or the Cr2–Cr2′–Cr1–SmA transitions of the 5TNCS_D compound along with the melting of PCL (Figure 7d). The anomaly associated with PCL melting undergoes a slight shift toward lower temperatures (328.3–328.1 K) in fibers containing 5TNCS_H (Figure 7a), whereas the presence of 5TNCS_D results in a more pronounced decrease in PCL melting temperature (325.3–324.7 K) (Figure 7c). The reduction in melting temperature upon addition of the deuterated liquid crystal arises from changes in the strength and nature of interfacial interactions between the liquid crystal and PCL due to deuteration, which disrupts the crystallinity of PCL. This observation is consistent with a slightly weaker LC–PCL interaction in the deuterated system, which can be attributed to reduced polarizability and lower vibrational coupling at the interface. In contrast, the protonated liquid crystal exhibits stronger and more chemically compatible interactions with PCL, thereby preserving the crystalline structure to a greater extent and causing only minimal changes in the melting temperature.

In the PCL + 5TNCS_D fibers, the temperatures for the Cr2–Cr2′ (306.9–307.0 K) and Cr2′–Cr1 (321.0–322.3 K) phase transitions are higher than those observed for the 5TNCS_D in the bulk form (Table 2). In contrast, for the PCL + 5TNCS_H fibers, the Cr2–Cr1 transition temperatures (310.0–313.6 K) are lower compared to that of the bulk 5TNCS_H. However, the Cr1–SmA transition temperature (ca. 332 K) remains unchanged in both fiber systems relative to the bulk compounds. In the case of the deuterated liquid crystal, the elevated crystal–crystal transition temperatures observed in the fibers are attributed to geometric confinement and orientation-induced stresses within the fiber structure (in the presence of PCL), which stabilize specific crystalline phases of the liquid crystal and increase the energy barrier for phase transitions. Conversely, for the protonated liquid crystal, the opposite effect is observed. In the bulk state, the structures are more ordered and free from morphological constraints, facilitating phase reorganization. Within the fibers, however, strong interactions with PCL disrupt this order, resulting in a decrease in the transition temperature.

In terms of the enthalpy (Δ*H*) and entropy (Δ*S*) changes, phase transitions between crystalline phases in the PCL-embedded fibers exhibit higher values compared to the bulk compounds (Table 2), whereas the opposite trend is observed for the Cr1–SmA transition. In the bulk state, the Cr2 to Cr1 transition involves only subtle rearrangements within partially disordered crystalline phases and is therefore associated with very small thermodynamic changes. Under polymer confinement, however, this transition additionally involves the relaxation of orientation-induced stresses, restructuring of confined liquid crystal domains, and modifications of liquid crystal–polymer interfacial interactions, leading to significantly enhanced Δ*H* and Δ*S* values. In contrast, the enthalpy and entropy changes associated with the Cr1–SmA transition are strongly reduced in the fibers, indicating that the development of smectic order is partially constrained or preorganized by the fiber geometry and the presence of the polymer matrix, which limits the extent of structural reorganization during the transition. Moreover, the enthalpy and entropy changes for deuterated 5TNCS_D are generally slightly lower than those of 5TNCS_H, consistent with the isotope effect, as the heavier deuterium atoms reduce vibrational amplitudes and overall molecular mobility within the liquid-crystalline phases.

### 3.3 Molecular Dynamics of 5TNCS_X in the Bulk Form

The dynamics of relaxation in the various thermodynamic states are described by using BDS results. The 5TNCS_H forms N, SmA, two crystalline Cr1 and Cr2 phases, and a glassy gCr2 state, and the 5TNCS_D additionally exhibits the cold crystallization process. Figure 8a,b shows the dielectric loss (ε″) as a function of frequency at selected temperatures for both isotopologues.

**Figure 8:**
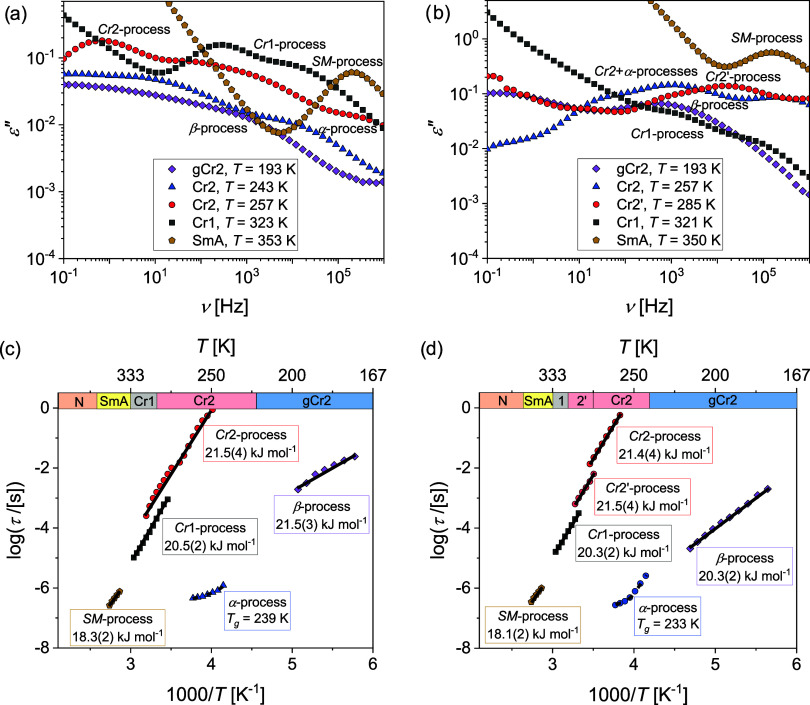
Dielectric loss ε″ vs frequency of relaxation processes at selected temperatures (a, b) and the temperature dependence of the relaxation times τ (c, d) of the 5TNCS_H (a, c) and 5TNCS_D (b, d). Solid lines represent Arrhenius fits, and dashed lines correspond to VFT fits (c, d).

In the nematic phase, relaxation processes related to molecular rotations around the short axis (so-called low-frequency processes) and the long axis (so-called high-frequency processes) can typically be observed. Generally, the low-frequency process occurs in the megahertz range, while the high-frequency process occurs in the gigahertz range. However, in this case, the measurement range is too narrow, and the relaxation processes that might occur in the nematic phase likely fall outside the measurement window. In the SmA phase, a single high-frequency process is present (yellow curves in Figure 8a,b), known as the soft mode (SM), which involves the collective oscillation of the tilt angle of molecules. The temperature dependences of the relaxation times for the SM-process follow the Arrhenius equation, with an activation energy of ca. 18 kJ mol^–1^. In the crystalline phase, relaxation processes are usually not observed. However, due to the molecular structure of the studied compound, particularly the presence of fluorine atoms,^36^ a relaxation process is possible in the crystalline phases. The shape of the dielectric spectra in the Cr1 (gray curves in Figure 8a,b), as well as Cr2 and Cr2′ (red and blue curves in Figure 8a,b) phases, indicates that the transition between the crystalline phases is smooth. The Cr1-process, characteristic of the high-temperature crystalline phase Cr1, persists near the Cr2–Cr1 transition in the low-temperature crystalline phase Cr2, while a distinct Cr2-process appears in the Cr2 phase. For 5TNCS_D, there is also the Cr2′-process visible in the Cr2′ phase after cold crystallization. The temperature dependence of relaxation times for all processes in the crystalline phases is linear, and the activation energies are ca. 20 kJ mol^–1^ for the Cr1-processes, 19 kJ mol^–1^ for the Cr2′-process, and 21 kJ mol^–1^ for the Cr2-processes. During heating of the samples from the glassy state, two processes are visible: a secondary β-process at low temperatures (purple curves in Figure 8a,b) and a structural α-process at higher temperatures (blue curves in Figure 8a,b).

The relaxation times of these processes are determined by fitting the experimental complex permittivity in the frequency domain using the Havriliak–Negami (HN) equation^37^

6 $$\varepsilon^{*}\left(\omega \right)=\varepsilon^{\prime }\left(\omega \right)-i\varepsilon^{\prime \prime }\left(\omega \right)=\varepsilon_{\infty }+\sum_{k}\frac{\Delta \varepsilon_{k}}{{\left[1+{\left(i\omega \tau_{k}\right)}^{\alpha_{k}}\right]}^{\beta_{k}}}+\frac{i\sigma_{0}}{\varepsilon_{0}\omega^{n}}$$

where ε_*∞*_ is the permittivity at the high-frequency limits, Δε_*k*_ is the dielectric strength of the *k*-th process, τ_*k*_ is the dielectric relaxation time of the *k*-th dynamic process, σ_0_ is the electric conductivity, ε_0_ is the permittivity of the vacuum, and α_*k*_ and β_*k*_ are shape parameters describing the symmetric and asymmetric broadening of the loss spectra, respectively. For α*_k_* = 1 and β_*k*_ = 1, the relaxation process is of the Debye type, for 0 < α*_k_* < 1 and β_*k*_ = 1, it is the Cole–Cole type, and for 0 < α*_k_* < 1 and 0 < β*_k_* < 1, it is the HN type. One deals with the ohmic electric conductivity independent of frequency for *n* = 1, and then the quantity σ_0_ is the DC electric conductivity.^38^ For 0 < *n* < 1, one deals with the nonohmic electric conductivity, and then σ_0_ is the quantity proportional to the AC electric conductivity.^39^

Figure 8c,d presents the relaxation times as a function of temperature in the various thermodynamic phases of the 5TNCS_H and 5TNCS_D. The relaxation times of the β-processes change linearly with temperature, and their behavior is well described by the Arrhenius equation. By plotting the relationship between log τ and 1000/*T*, the activation energy *E*_A_ can be calculated from the slope of the resulting straight line, incorporating the gas constant value. The activation energy for the β-processes of both compounds is 31 kJ mol^–1^. The Ngai and Capaccioli formula allows us to verify if the β-process is the Johari–Goldstein (JG) relaxation^40^

7 $$\frac{E_{A,\beta }}{RT_{g}}=2.303\left[2-13.7\left(1-\beta_{\mathrm{KWW}}\right)-\log\tau_{\infty ,\beta }\right]$$

where β_KWW_ is the stretch parameter of the Kohlrausch–Williams–Watts (KWW) formula^41^ used to fit the α-loss peak (here β_KWW_ = 0.43 for all spectra). Kudlik et al.^42^ proved that $\frac{E_{A,\beta }}{RT_{g}}=24$ for the β-process classified as the JG mode in many glass formers. For both isotopologues, the equality of eq 7 is not maintained, so the β-relaxation is not of the JG type. The temperature dependence of the α-relaxation times τ_α_ is described by the Vogel–Fulcher–Tammann (VFT) formula^43^

8 $$\tau_{\alpha }=\tau_{\infty }\exp\left(\frac{D_{f}T_{0}}{T-T_{0}}\right)$$

where τ_∞_ is the preexponential factor, *D*_f_ is a constant parameter, and *T*_0_ is the Vogel temperature. The glass transition temperature *T*_g_ = 239 K for 5TNCS_H and *T*_g_ = 233 K for 5TNCS_D is defined as the temperatures at which τ_α_ = 100 s.

The relaxation times of the *Cr*-processes, as described by the HN model, follow the Arrhenius equation with activation energies indicated in Figure 8c,d, implying that they originate from intermolecular stochastic motions. DFT calculations to perform the potential energy scans for changing the torsional angles between the pentyl group and the molecular core are described in ref 2. The energy barrier for the rotation of the pentyl chain is 10.5 kJ mol^–1^. The two internal dihedrals of the *para*-terphenyl are coupled in the same manner, resulting in a total torsional potential energy of 18 kJ mol^–1^.^44^ By correlating the results of quantum chemical calculations with activation energy values for processes in crystalline phases, we hypothesize that each process may be associated with one of two possible scenarios: (*i*) the changing of rotations of the pentyl chain coupled with the torsional angles in the molecular core, and (*ii*) the rotation in the molecular core.

### 3.4 Molecular Dynamics of 5TNCS_X Embedded within Electrospun Fibers

In order to explore the molecular dynamics of the two-dimensionally confined systems, broadband dielectric spectroscopy is utilized. The dielectric spectra as a function of temperature (Figure 9a,b) and the resulting activation plots (Figure 9c,d) for the PCL + 5TNCS_X fibers, with relaxation times extracted from Havriliak–Negami fits of the complex permittivity spectra, are presented. For both compounds, 5TNCS_H and 5TNCS_D, studied in bulk form at temperatures below 300 K, two relaxation processes are observed. At higher temperatures, a structural α-process appears, while at lower temperatures, a secondary β-process is visible. In the case of pure PCL fibers, the segmental α-process and the local β-process are also observed, which are associated with the motions of the polar ester group within the repeat unit [-(CH_2_)_5_COO-]. In contrast, the PCL + 5TNCS_X fibers exhibit three relaxation processes: two of the α-type and one of the β-type.

**Figure 9:**
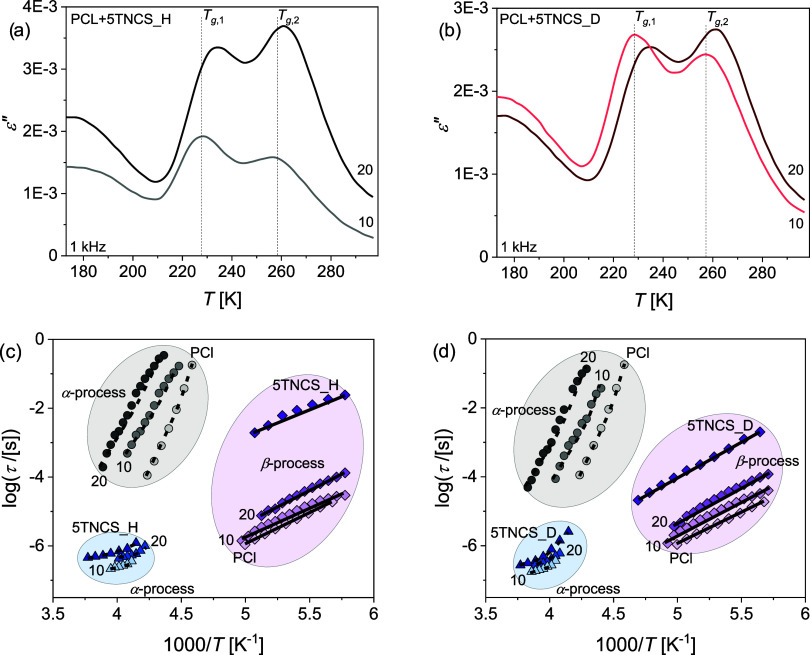
Comparison of dielectric loss spectra for frequency 1 kHz for PCL+5TNCS_H (a) and 5TNCS_D (b) fibers. Thermal activation plots of structural α-processes and secondary β-processes for PCL + 5TNCS_H (c) and PCL+5TNCS_D (d) fibers compared to the PCL fiber and bulk liquid crystals upon heating. Solid lines represent Arrhenius fits, and dashed lines correspond to VFT fits (c, d).

The calculated glass transition temperatures and activation energy values are summarized in Table 3. For PCL fibers without the liquid crystal additive, the obtained glass transition temperature *T*_g,1_ is approximately 209 K. The incorporation of the liquid crystal leads to an increase in *T*_g,1_, reaching 227–235 K for 5TNCS_H and 228–234 K for 5TNCS_D. This increase in *T*_g,1_ is more pronounced in the results obtained from BDS than from DSC experiments. A similar effect is observed for the second glass transition temperature *T*_g,2_, which shifts within the range of 257–262 K with increasing content of 5TNCS_H in the fibers, and within the range of 256–261 K with increasing content of 5TNCS_D. For pure PCL fibers, the activation energy of the β-process is approximately 34.6 kJ mol^–1^ and decreases with increasing concentration of the liquid crystal: ranging from 33.5 to 32.3 kJ mol^–1^ for 5TNCS_H, and from 33.8 to 32.7 kJ mol^–1^ for 5TNCS_D. The β-process is associated with local (dipolar) molecular mobility. The observed decrease in activation energy of the β-process with increasing liquid crystal content can be interpreted as a result of enhanced local molecular mobility, attributed to an increase in free volume and a relaxation of local intermolecular interactions. Interestingly, the magnitude of this decrease is smaller for the deuterated sample, implying that isotopic substitution locally stiffens the potential energy surface and reduces the amplitude of the dipolar fluctuations.

**Table 3:** Calculated Dielectric Glass Transition Temperatures (*T*_g,1_ and *T*_g,2_) for Both α-Processes from the VFT Equation and the Obtained Activation Energies (*E*_A_) for β-Process from the Arrhenius Equation

sample	*T*_g,1_ [K]	*T*_g,2_ [K]	*E*_A_ of β-process [kJ mol^–1^]
PCL	209		34.6
PCL + 10 mg 5TNCS_H	227	257	33.5
PCL + 20 mg 5TNCS_H	235	262	32.2
5TNCS_H (bulk)		239	31.0
PCL + 10 mg 5TNCS_D	228	256	33.8
PCL + 20 mg 5TNCS_D	234	261	32.7
5TNCS_D (bulk)		233	31.0

The results obtained from DSC and BDS studies clearly indicate the complex molecular nature of PCL fibers containing the liquid crystal 5TNCS_X. The observation of two glass transition temperatures, along with the presence of two α-relaxations and one β-relaxation, demonstrates that the material structure is not homogeneous, but instead consists of distinct domains exhibiting different segmental and local mobility. An increase in the liquid crystal content induces changes in both the crystalline structure and molecular mobility. The incorporation of 5TNCS_X disrupts local ordering, increases the free volume, and consequently reduces the activation energy of the β-relaxation process. This effect can be explained by the presence of microdomains with enhanced dynamic freedom, where liquid crystal molecules hinder the tight packing of PCL segments. This phenomenon of microheterogeneity in the studied system is manifested not only through the separation of glass transition temperatures but also through changes in the dielectric response, which is characteristic of materials exhibiting nanoscale phase separation. Furthermore, it was observed that increasing the concentration of 5TNCS_X leads to a shift of *T*_g,2_ toward lower temperatures, suggesting increased mobility within the domains enriched in the liquid crystal.

### 3.5 Comparison of Isotopic and Confinement Effects

A direct comparison between the protonated (5TNCS_H) and deuterated (5TNCS_D) compounds, as well as between bulk and fiber forms, reveals several systematic and physically meaningful trends.

In the bulk state, both isotopologues exhibit similar mesomorphic phase sequences and closely related transition temperatures, confirming that deuteration does not significantly alter the overall thermodynamic stability of the liquid-crystalline phases. However, a qualitative difference emerges in the glassy regime: cold crystallization occurs exclusively in 5TNCS_D, while it is absent in the protonated analogue. This behavior demonstrates that deuteration subtly modifies the free-energy landscape, reducing molecular mobility in the glassy state and thereby suppressing crystallization during cooling but enabling diffusion-controlled nucleation upon reheating. The higher molecular mobility of 5TNCS_H prevents such delayed crystallization, highlighting the sensitivity of the kinetic pathways to isotopic substitution.

Dielectric spectroscopy further reveals that deuteration affects relaxation dynamics on the molecular scale. While the general character of α- and secondary relaxation processes is preserved, the deuterated system exhibits slightly higher activation energies and reduced relaxation strength, indicating a locally stiffer potential energy surface and diminished dipolar fluctuations. These effects are consistent with isotope-induced changes in vibrational dynamics and zero-point energies, which primarily influence local motions rather than global phase stability.

When the system is transferred from bulk to confined fiber geometry, confinement introduces an additional level of complexity. In the fibers, both isotopologues show systematic shifts of glass transition temperatures toward higher values compared to the bulk, reflecting restricted chain mobility and enhanced interfacial interactions with the polymer matrix. Polymer confinement redistributes the enthalpy between different phase transitions rather than conserving bulk-like thermodynamic behavior. Confinement also leads to a modification of relaxation behavior, manifested by changes in activation energies and relaxation times, particularly for secondary processes associated with local molecular mobility.

Importantly, the effects of confinement are not identical for the two isotopologues. The deuterated compound exhibits a smaller reduction in activation energy for the β-process upon confinement, indicating that isotopic substitution partially counteracts the mobility-enhancing effect of the free volume and interfaces. This demonstrates that confinement and isotopic substitution act in a nonadditive manner, jointly shaping molecular dynamics through competing influences on free volume, intermolecular interactions, and segmental flexibility.

## 4 Conclusions

Isotopic substitution and spatial confinement jointly control the crystallization and relaxation dynamics of partially fluorinated terphenyls. Both protonated and deuterated compounds exhibit comparable mesophases, but only the deuterated variant undergoes cold crystallization, reflecting altered thermodynamic pathways due to isotope effects.

The presence of two distinct glass transition temperatures in PCL fibers modified with liquid crystals (5TNCS_H or 5TNCS_D) suggests a phase-separated microstructure comprising domains with different molecular mobility. From an application standpoint, this microphase heterogeneity can be advantageous in the design of advanced functional materials. The lower-*T*_g,1_ domain, rich in semicrystalline PCL, contributes to mechanical integrity and thermal stability, while the higher-*T*_g,2_ domain, enriched in the liquid crystal, offers increased local chain mobility. This combination allows for the development of materials that are simultaneously robust and dynamically responsive. Such properties are particularly attractive for applications in flexible electronics, smart textiles, or soft actuators, where controlled viscoelastic behavior, shape adaptability, or stimuli-responsiveness are required. Furthermore, the ability to tune the ratio and mobility of these domains, through the type and concentration of the liquid crystal, provides a pathway to engineer the thermal, mechanical, and dielectric performance of fiber-based systems for specific functional requirements. Future work combining dielectric spectroscopy with neutron or X-ray scattering could further clarify the molecular mechanisms driving isotopic- and confinement-induced modifications in molecular dynamics.

## Supplementary Material



## Notes

Phase transition temperatures and thermodynamic functions of 5TNCS_X compounds (PDF)
