# Supplementary material for: Isotopic and Confinement Effects on the Phase Behavior and Molecular Dynamics of Protonated and Deuterated Terphenyls in Electrospun Fibers
Source: J Phys Chem B. 2026 Feb 20;130(9):2676–88. doi: 10.1021/acs.jpcb.5c07839 (PMC13299012; doi:10.1021/acs.jpcb.5c07839)
Supplement: Supplementary file 1 [file jp5c07839_si_001.pdf]

Supporting Information

Isotopic and confinement effects on the phase behaviour and molecular dynamics of protonated and deuterated terphenyls in electrospun fibres

Anna Drzewicz[a], Szymon Folta[b], Olga Adamczyk[a], Marta Żak[c] and Ewa Juszyńska-Gałązka[a,d]*

[a]Institute of Nuclear Physics Polish Academy of Sciences, PL-31342 Krakow, Poland

[b]Faculty of Materials Science and Physics, Cracow University of Technology, PL-30084 Krakow, Poland

[c]Institute of Chemistry, Military University of Technology, PL-00908 Warszawa, Poland

[d]Research Center for Thermal and Entropic Science, Graduate School of Science, Osaka University, 560-0043 Osaka, Japan

Table S1. Phase transition temperatures (onset temperature T_{onset} and peak temperature T_{peak}) and thermodynamic functions (enthalpy change ΔH and entropy change ΔS) during cooling and heating of the 5TNCS_H.

cooling								
	Iso-N				N-SmA			
$\Delta T/\Delta t$ [K min^{-1}]	T_{onset} [K]	T_{peak} [K]	ΔH [kJ mol^{-1}]	ΔS [J mol^{-1} K^{-1}]	T_{onset} [K]	T_{peak} [K]	ΔH [kJ mol^{-1}]	ΔS [J mol^{-1} K^{-1}]
2	455.05	455.00	0.77	1.70	372.31	371.77	0.11	0.31
5	455.02	454.93	0.96	2.12	372.24	371.91	0.18	0.48
10	454.86	454.68	0.96	2.10	372.44	372.07	0.16	0.44
15	454.85	454.53	1.06	2.34	372.02	371.75	0.17	0.45
20	454.74	454.38	1.05	2.32	372.17	371.81	0.15	0.39
25	454.71	454.21	1.09	2.39	371.93	371.37	0.14	0.37
30	454.62	454.03	1.13	2.49	372.03	371.44	0.14	0.37
	SmA-Cr2				Cr2-gCr2			
$\Delta T/\Delta t$ [K min^{-1}]	T_{onset} [K]	T_{peak} [K]	ΔH [kJ mol^{-1}]	ΔS [J mol^{-1} K^{-1}]	T_g [K]			
2	303.04	302.35	17.09	56.53	242.05			
5	300.78	300.82	16.49	54.81	242.85			
10	297.50	296.78	16.01	53.96	239.91			
15	297.67	295.32	15.29	51.76	240.84			
20	297.21	296.04	15.60	52.71	240.74			
25	295.80	290.24	14.51	50.01	240.98			
30	296.55	294.16	15.23	51.79	240.99			
heating								
	Cr2-Cr1				Cr1-SmA			
$\Delta T/\Delta t$ [K min^{-1}]	T_{onset} [K]	T_{peak} [K]	ΔH [kJ mol^{-1}]	ΔS [J mol^{-1} K^{-1}]	T_{onset} [K]	T_{peak} [K]	ΔH [kJ mol^{-1}]	ΔS [J mol^{-1} K^{-1}]
2	318.43	319.39	0.06	0.17	330.32	331.27	19.73	59.56
5	318.28	319.51	0.04	0.13	330.39	331.51	19.46	58.69
10	316.97	317.08	0.13	0.41	330.54	331.80	19.94	60.08
15	318.70	319.64	0.07	0.21	328.96	331.71	20.09	60.56
20	318.51	319.88	0.04	0.14	330.44	332.11	20.38	61.36
25	318.89	319.71	0.03	0.08	329.06	332.03	18.90	56.92
30	318.80	320.21	0.05	0.17	330.63	332.42	19.30	58.07
	SmA-N				N-Iso			
$\Delta T/\Delta t$ [K min^{-1}]	T_{onset} [K]	T_{peak} [K]	ΔH [kJ mol^{-1}]	ΔS [J mol^{-1} K^{-1}]	T_{onset} [K]	T_{peak} [K]	ΔH [kJ mol^{-1}]	ΔS [J mol^{-1} K^{-1}]
2	371.64	372.39	0.11	0.29	455.19	455.28	1.61	3.55
5	371.91	372.67	0.27	0.72	455.24	455.39	1.72	3.78
10	372.66	372.95	0.18	0.48	455.25	455.47	1.80	3.95
15	372.37	372.74	0.13	0.36	455.37	455.71	1.55	3.40
20	372.50	373.27	0.14	0.38	455.38	455.81	1.76	3.87

25	372.40	373.12	0.10	0.26	455.45	456.04	2.08	4.56
30	372.23	373.45	0.24	0.64	455.47	456.15	2.61	5.73

Table S2. Phase transition temperatures (onset temperature T_{onset} and peak temperature T_{peak}) and thermodynamic functions (enthalpy change ΔH and entropy change ΔS) during cooling and heating of the 5TNCS_D.

cooling								
	Iso-N				N-SmA			
$\Delta T/\Delta t$ [K min^{-1}]	T_{onset} [K]	T_{peak} [K]	ΔH [kJ mol^{-1}]	ΔS [J mol^{-1} K^{-1}]	T_{onset} [K]	T_{peak} [K]	ΔH [kJ mol^{-1}]	ΔS [J mol^{-1} K^{-1}]
2	455.54	455.68	0.88	1.94	369.3	369.97	0.09	0.25
5	455.43	455.58	1.41	3.09	369.41	369.96	0.15	0.40
10	454.79	455.06	1.29	2.84	369.49	369.84	0.12	0.31
15	454.79	455.18	1.27	2.78	369.56	369.85	0.12	0.32
20	454.71	455.19	1.35	2.96	369.41	369.71	0.1	0.26
25	454.57	455.17	1.32	2.91	369.04	369.5	0.06	0.15
30	454.33	455.07	1.46	3.21	368.96	369.46	0.07	0.19
	SmA-Cr2				Cr2-gCr2			
$\Delta T/\Delta t$ [K min^{-1}]	T_{onset} [K]	T_{peak} [K]	ΔH [kJ mol^{-1}]	ΔS [J mol^{-1} K^{-1}]	T_g [K]			
2	301.98	303.05	16.82	55.66	237.52			
5	300.53	301.62	16.68	55.46	237.84			
10	298.22	299.93	16.10	53.97	233.27			
15	296.80	299.32	15.87	53.44	233.19			
20	295.61	298.70	15.58	52.68	233.34			
25	294.09	297.22	15.27	51.89	233.04			
30	292.66	296.8	14.76	50.42	233.27			
heating								
	Cr2-Cr2’				Cr2’-Cr1			
$\Delta T/\Delta t$ [K min^{-1}]	T_{onset} [K]	T_{peak} [K]	ΔH [kJ mol^{-1}]	ΔS [J mol^{-1} K^{-1}]	T_{onset} [K]	T_{peak} [K]	ΔH [kJ mol^{-1}]	ΔS [J mol^{-1} K^{-1}]
2	287.06	291.96	0.30	1.03	317.73	318.57	0.02	0.05
5	291.24	295.89	0.33	1.12	317.96	318.98	0.02	0.06
10	292.88	298.19	0.51	1.70	318.02	318.52	0.02	0.06
15	295.39	300.67	1.01	3.36	318.21	318.91	0.01	0.02
20	297.87	300.74	1.08	3.58	318.52	319.04	0.03	0.09
25	296.38	302.63	1.09	3.59	318.37	319.37	0.04	0.12
30	298.85	304.48	1.17	3.84	318.71	319.45	0.03	0.09
	Cr1-SmA				SmA-N			
$\Delta T/\Delta t$ [K min^{-1}]	T_{onset} [K]	T_{peak} [K]	ΔH [kJ mol^{-1}]	ΔS [J mol^{-1} K^{-1}]	T_{onset} [K]	T_{peak} [K]	ΔH [kJ mol^{-1}]	ΔS [J mol^{-1} K^{-1}]
2	330.17	331.40	18.59	56.07	369.28	369.91	0.11	0.29
5	330.22	331.67	18.86	56.83	370.14	370.30	0.16	0.42
10	330.27	331.99	19.00	57.21	370.45	370.82	0.14	0.39

15	330.38	332.24	17.73	53.35	370.60	370.96	0.08	0.21
20	330.51	332.47	18.70	56.23	370.64	371.06	0.07	0.19
25	330.57	332.65	18.58	55.84	370.38	371.15	0.15	0.39
30	330.73	332.89	18.61	55.87	370.45	371.39	0.19	0.51
	N-Iso							
$\Delta T/\Delta t$ [K min^{-1}]	T_{onset} [K]	T_{peak} [K]	ΔH [kJ mol^{-1}]	ΔS [J mol^{-1} K^{-1}]				
2	455.88	456.00	1.65	3.62				
5	455.88	456.07	1.57	3.43				
10	455.79	456.06	1.60	3.51				
15	455.97	456.37	0.17	3.62				
20	456.09	456.63	1.97	4.31				
25	456.22	456.86	1.63	3.58				
30	456.24	457.02	1.99	4.35				

The ΔS values reported for non-equilibrium transitions are intended as comparative indicators rather than exact thermodynamic quantities.
